# Retroperitoneoscopic bladder neck closure for continuous urinary incontinence in Fournier's gangrene

**DOI:** 10.1002/iju5.12788

**Published:** 2024-09-20

**Authors:** Yukiko Doi, Atsuhiko Ochi, Yuma Okamoto, Akira Komiya, Hiroshi Kuji, Koichiro Suzuki, Naoki Shiga, Hirokazu Abe

**Affiliations:** ^1^ Department of Urology Kameda Medical Center Kamogawa City Chiba Japan; ^2^ Department of Urology The Jikei University School of Medicine Tokyo Japan

**Keywords:** bladder neck closure, catheterization, Fournier gangrene, Retroperitoneoscopic surgery, urinary incontinence

## Abstract

**Introduction:**

A retroperitoneoscopic procedure for bladder neck closure has not yet been described.

**Case presentation:**

Case 1 was a 56‐year‐old man who underwent clean intermittent catheterization for spastic paraplegia due to a thoracic spinal cord injury 37 years prior. Case 2 was an 80‐year‐old bedridden woman who underwent urethral catheterization after a femoral fracture and brain infarction 3 years prior. Both patients presented with Fournier's gangrene, and the urogenital region, including the necrotic urethra, was debrided. Although a permanent suprapubic catheter was established, postoperative wound healing was poor owing to continuous urine leakage from the urethral stump. Therefore, we performed retroperitoneoscopic bladder neck closure, and the urinary incontinence was completely resolved.

**Conclusion:**

Retroperitoneoscopic bladder neck closure is a feasible and less invasive procedure for treating continuous urinary incontinence from the urethral stump in patients with Fournier's gangrene after surgical debridement and having a permanent suprapubic catheter.

**Keynote message**

Long‐term management of a urethral catheter for a neurogenic bladder is a risk factor for Fournier's gangrene. When continuous urine leakage from the urethral stump occurs after debridement and suprapubic catheterization, postoperative wound healing is poor. In such cases, retroperitoneoscopic bladder neck closure is a feasible and less invasive procedure for resolving urinary incontinence.

**Abbreviations & Acronyms**
Bl: bladder
FG: Fournier's gangrene
Pr: prostate
SPC: suprapubic catheter
Ur: urethra

## Introduction

FG is an acute and fatal necrotizing fasciitis of the urogenital, perineal, or perianal region. Owing to its high mortality rate, rapid debridement of necrotizing tissue and broad‐spectrum antibacterial treatment are required.^1^, ^2^ The wound is usually left open after the initial debridement.^3^ Additional resection and debridement are performed 3.5 times on average.^4^ When the soft tissue infection is resolved, the wound is closed with plastic surgery.^1^

When FG involves the urethra, all necrotic tissues, including urethral tissue, are excised, and suprapubic catheterization is performed to avoid prolonged infections due to bacterial colonization on the transurethral catheter.^2^ However, in cases of neurogenic bladder with low bladder capacity, detrusor dysfunction, or urethral sphincter deficiency, continuous urinary incontinence from the urethral stump may occur despite suprapubic catheterization, resulting in poor wound healing.^5^, ^6^ Although the effectiveness of open surgical bladder neck closure in such cases has been reported, a retroperitoneoscopic procedure has not yet been described.

Herein, we report our successful experiences with retroperitoneoscopic bladder neck closure for continuous urinary incontinence after debridement and suprapubic catheterization in two patients with FG.

## Case presentation

### Case 1

**Fig. 1:**
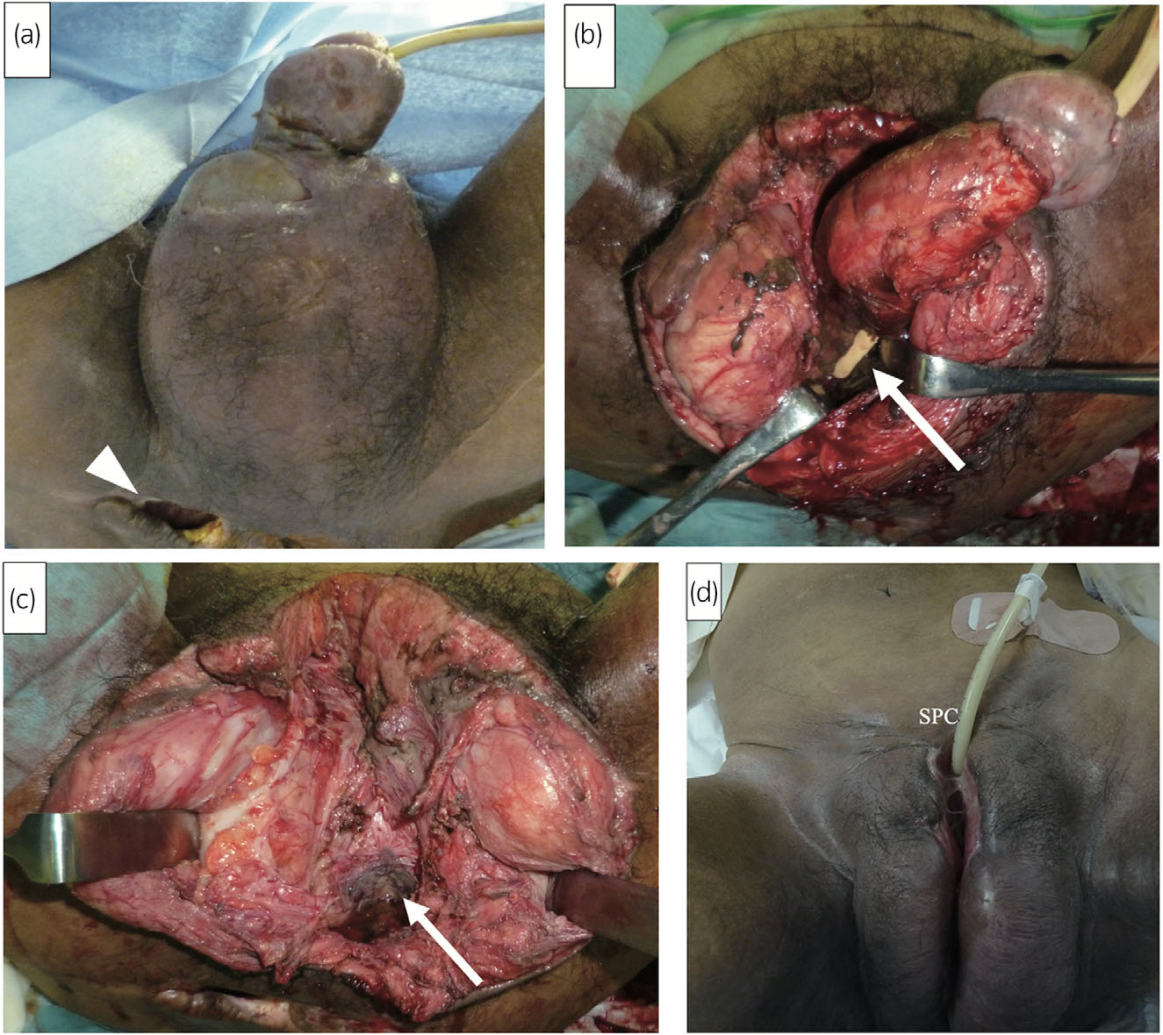
Surgical debridement for Fournier's gangrene in a 56‐year‐old man (Case 1). (a) The scrotum appears erythematic and swollen. The right ischial decubitus is shown (arrowhead). (b) Following debridement of the scrotum and bilateral orchiectomy, the bulbous urethra was necrotic (arrow) and contiguous to the ischial decubitus. (c) Following debridement of the penis, the urethra was resected as much as possible. The arrow indicates the urethral stump. (d) Six weeks after surgical debridement, the wound was not closed.

Owing to a fall 37 years earlier, a 56‐year‐old man sustained a thoracic spinal cord (T6) injury that resulted in spastic paraplegia, for which he underwent clean intermittent catheterization. He presented with FG in the urogenital region (Fig. 1a) and underwent emergent surgical debridement. The bulbous urethra was necrotic, and a fistula was observed in the ischial decubitus (Fig. 1b,c). The urethra was resected and sutured, and a SPC was established. Urine culture revealed polymicrobial patterns, including Methicillin‐resistant Staphylococcus aureus, Pseudomonas aeruginosa, Enterobacteriaceae species, as well as Bacteroides species, and multi‐antimicrobial therapy with Piperacillin‐tazobactam, Vancomycin, and *Clindamycin* are administered. However, continuous urinary incontinence from the urethral stump occurred postoperatively, resulting in poor wound healing (Fig. 1d). Therefore, we performed retroperitoneoscopic bladder neck closure 6 weeks after the surgical debridement.

**Fig. 2:**
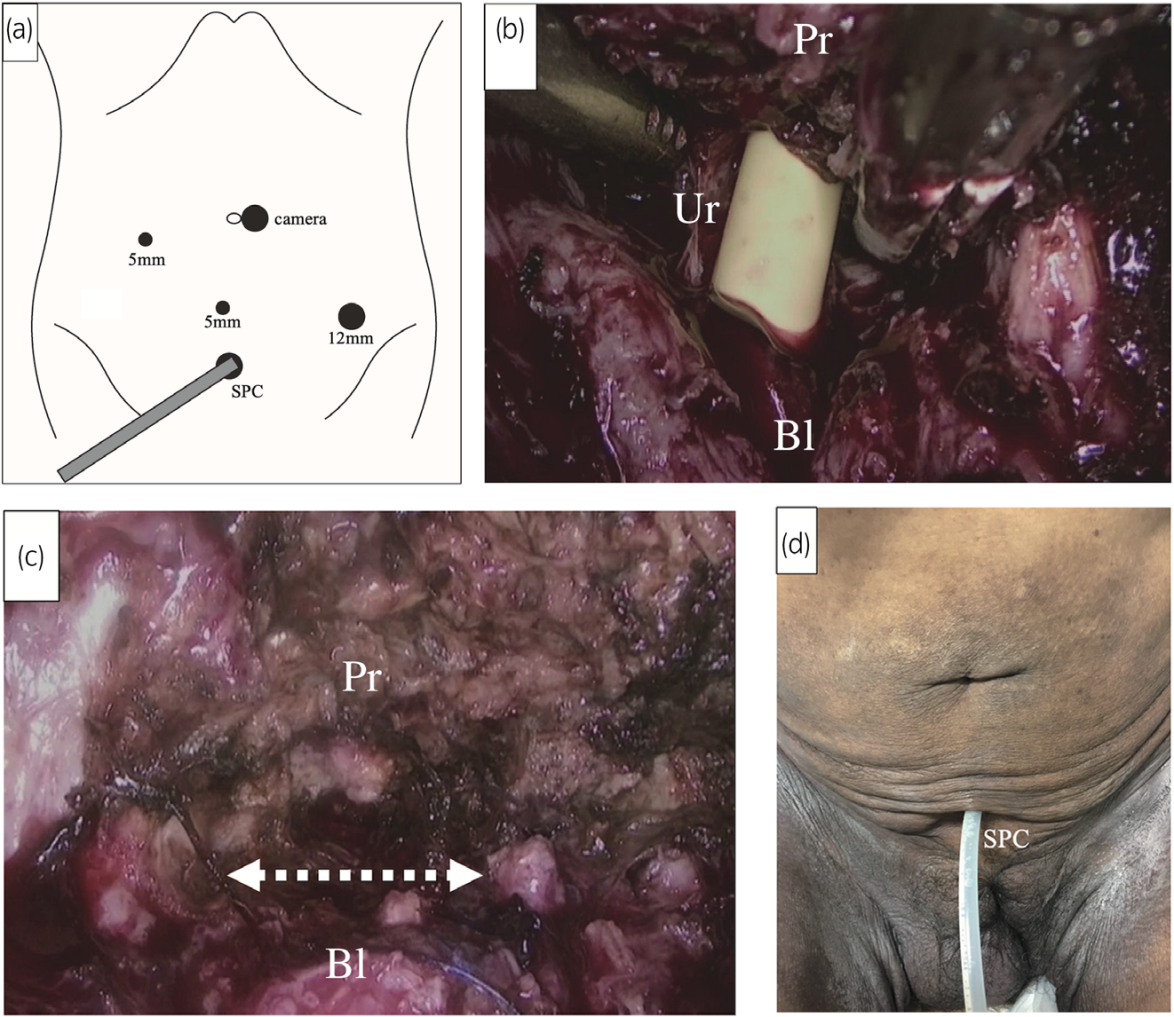
Retroperitoneoscopic bladder neck closure for a 56‐year‐old man (Case 1). (a) Schema of the trocars. (b) The urethra was cut at the internal urethral orifice. (c) Both the proximal urethra and bladder neck were sutured and closed. The bladder and prostate were completely separated at the line indicated by a two‐headed arrow. (d) The perineal wound was closed after the bladder neck closure.

Retroperitoneoscopic bladder neck closure was performed under general anesthesia with the patient in the supine open‐leg position. A Foley catheter was inserted into the urethral stump at the perineum. A small transverse incision was made on the left side of the umbilicus, followed by the insertion of a distension balloon and expansion of the retroperitoneum. Through the incision, a camera trocar port was inserted into the pneumoperitoneum. Using a 0° laparoscope, three additional ports were placed in the retroperitoneum (Fig. 2a). The adhesion around the SPC was resected. The area between the prostate and bladder was identified by referring to the position of the Foley catheter balloon, and the bladder neck was cut using a vessel‐sealing device (Fig. 2b). The urethra was sutured continuously with 3–0 braided absorbable sutures, and the bladder neck was sutured continuously in two layers with 3–0 and 2–0 braided absorbable sutures (Fig. 2c). The total surgical time was 3 h 30 min, and the estimated blood loss was 50 mL. After surgery, urine leakage completely stopped, and wound closure and healing were achieved (Fig. 2d).

### Case 2

**Fig. 3:**
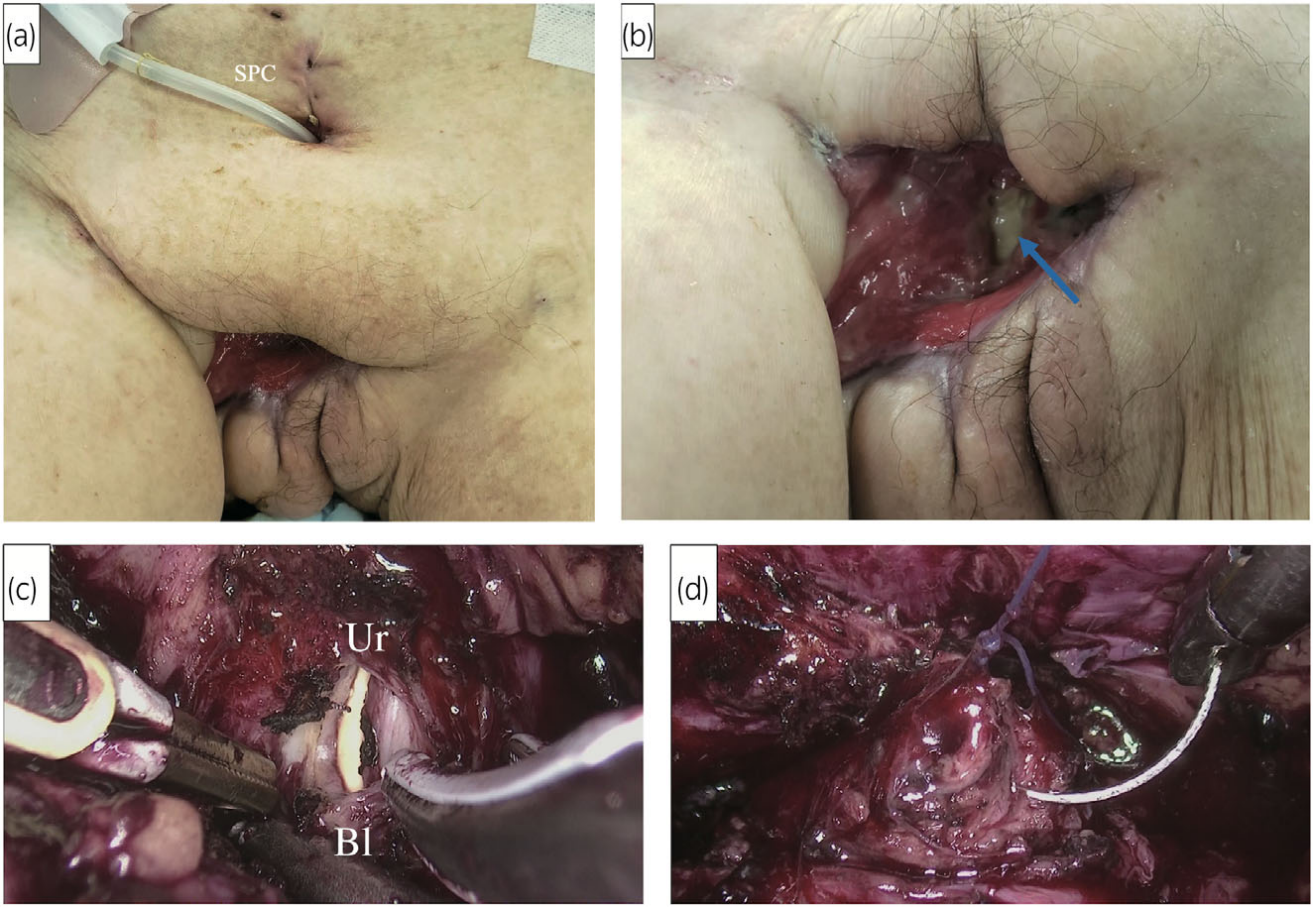
Retroperitoneoscopic bladder neck closure for an 80‐year‐old woman (Case 2). (a) Ten weeks after surgical debridement, the wound was not closed. (b) The arrow indicates the stump of the urethra. (c) The urethra was cut at the internal urethral orifice. (d) Both the proximal urethra and bladder neck were sutured and closed.

An 80‐year‐old woman with schizophrenia and diabetes mellitus bedridden after a femoral fracture and brain infarction underwent permanent bladder catheterization 3 years previously. Following the presentation at our hospital, she was transferred to the emergency department because of FG in the urogenital region. She underwent emergent debridement of the urogenital region, including part of the urethra, and a SPC was placed. Urine culture revealed multi‐species of gram‐positive coccus and rods, as well as gram‐negative rods and Bacteroides, and multi‐antimicrobial therapy with Piperacillin‐tazobactam, Vancomycin, and *Clindamycin* is performed. However, continuous urinary incontinence from both the urethral stump and urethrovaginal fistula remained (Fig. 3a,b). Owing to prolonged failure in wound healing, we performed laparoscopic bladder neck closure 10 weeks after the surgical debridement. The bladder neck was cut between the urethra and bladder, followed by closure as in case 1 (Fig. 3c,d). The total surgical time was 2 h 45 min, with an estimated blood loss of 10 mL, and urine leakage disappeared after surgery.

## Discussion

FG is a necrotizing fasciitis around the perineum, which requires emergency excision of necrotic tissues and appropriate antimicrobial therapy to save life. However, urine leakage from the urethral stump after debridement may cause delayed wound healing. Herein, two cases of continuous urinary incontinence from the urethral stump after debridement and suprapubic catheterization for FG were successfully treated by retroperitoneoscopic bladder neck closure.

**Fig. 4:**
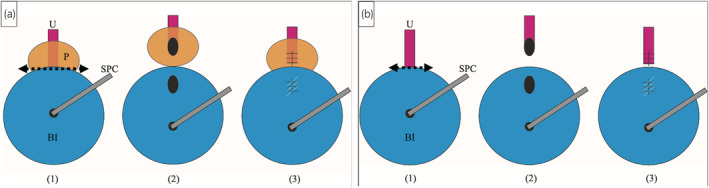
(a) Schema of the laparoscopic bladder neck closure for men. (1) The bladder neck is cut between the prostate and the bladder (two‐headed arrow). (2) The urethra and bladder are completely separated from each other at the internal urethral orifice. (3) The urethra and bladder neck are sutured and closed. (b) Schema of the laparoscopic bladder neck closure for women. (1) The bladder neck is cut between the urethra and the bladder (two‐headed arrow). (2) The urethra and bladder are completely separated from each other at the internal urethral orifice. (3) The urethra and bladder neck are sutured and closed.

Bladder neck closure with permanent suprapubic catheterization is a viable treatment option for intractable urinary incontinence due to a neurogenic bladder.^7^, ^8^ In patients with spinal cord injuries, this procedure stops urine leakage from the urethrocutaneous fistula.^5^, ^6^ However, in previous reports of bladder neck closure, open surgery was performed, whereas in our two cases, retroperitoneoscopic surgery with minimal incisions was performed. In open surgery reported previously, the anterior bladder wall was widely cut open, and the bilateral ureteral orifices were cannulated to avoid injury.^6^, ^7^, ^8^ By making it easier to approach the bladder neck more distally, our procedure allows surgeons to cut directly at the internal urethral orifice, which eliminates the risk of injury to the ureteral orifice and the need for ureteral catheterization, even in patients with an atrophic bladder. To prevent postoperative bladder neck fistulas, the omentum, rectus muscle flap, or other tissues can be interposed between the bladder neck and urethra.^8^ However, this is unnecessary when our technique is performed because the bladder opening can be minimized. Robotic‐assisted laparoscopic bladder neck closure has also been performed in recent years in pediatric surgery.^9^ Our retroperitoneoscopic surgery is achievable even after suprapubic catheterization by resection of the adhesions surrounding the catheter and the risk of intestinal injury is low. This procedure is feasible in both men (Fig. 4a) and women (Fig. 4b).

In conclusion, retroperitoneoscopic bladder neck closure for continuous urinary incontinence after debridement and suprapubic catheterization for FG is feasible and less invasive.

## Author contributions

Yukiko Doi: Project administration; writing – review and editing. Atsuhiko Ochi: Methodology; writing – original draft. Yuma Okamoto: Resources. Akira Komiya: Resources. Koichiro Suzuki: Supervision. Hiroshi Kuji: Resources. Hirokazu Abe: Supervision. Naoki shiga: Supervision.

## Conflict of interest

The authors declare no conflicts of interest.

## Informed consent

Informed consent for publication was obtained from the families of both patients.

## Approval of the research protocol by an Institutional Review Board

Not applicable.

## Registry and Registration No. of the study/trial

Not applicable.
